# Crystallite Size Effects on the Heat of Water Intrusion/Extrusion into/from Metal–Organic Frameworks

**DOI:** 10.1021/acs.jpclett.4c02639

**Published:** 2025-02-20

**Authors:** Liam J. W. Johnson, Alexander R. Lowe, Andrea Le Donne, Emre Arkan, Sebastiano Merchiori, Luis Bartolomé, Eder Amayuelas, Diego Mirani, Gabriel A. López, Giulia Grancini, Mirosław Chora̧żewski, Simone Meloni, Yaroslav Grosu

**Affiliations:** † Centre for Cooperative Research on Alternative Energies (CIC energiGUNE), Basque Research and Technology Alliance (BRTA), Vitoria-Gasteiz 01510, Spain; ‡ Department of Physics, Faculty of Science and Technology, University of the Basque Country (UPV/EHU), Barrio Sarriena s/n, Bilbao 48490 Leioa, Spain; ¶ Institute of Chemistry, University of Silesia in Katowice, Szkolna 9, 40-006 Katowice, Poland; § Dipartimento di Scienze Chimiche e Farmaceutiche (DipSCF), Università degli Studi di Ferrara (Unife), Via Luigi Borsari 46, I-44121 Ferrara, Italy; ∥ Department of Chemistry and INSTM, University of Pavia, Via Taramelli 14, Pavia I-27100, Italy

## Abstract

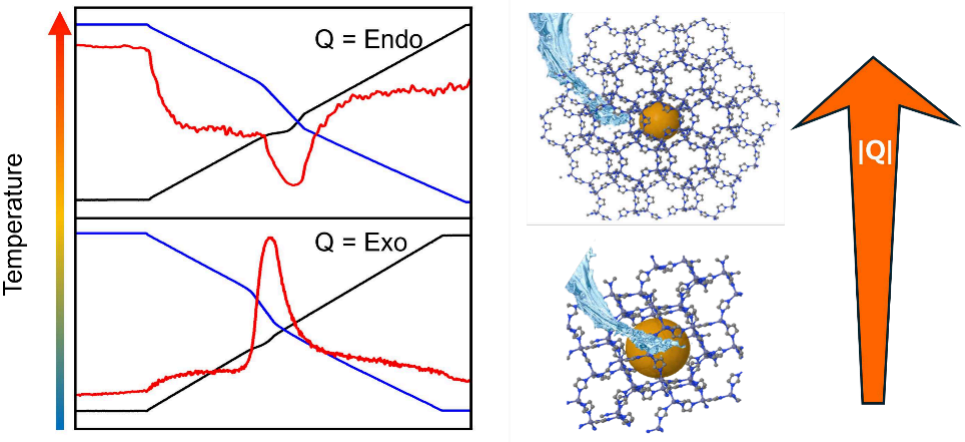

The wettability of nanoporous materials is a key property for a diverse range of applications. However, the heat generated in this process remains largely unexplored. Herein, the heats of intrusion/extrusion into/from ZIF-8 + water systems of various ZIF-8 crystallite sizes were measured at different temperatures. We found that decreasing crystallite size to the nanoscale resulted in a reduction of the magnitude of the heats of intrusion/extrusion. These results were mirrored in simulations, where the reduction of intrusion heat by reducing the characteristics dependent on crystallite size was comparable to the values obtained experimentally. We related this to the reduction in filling at lower pressures. We recorded the inversion of the sign of the heats of intrusion/extrusion measured at high temperatures. In addition, the heat/work ratio of the intrusion/extrusion processes was dependent on temperature while independent of crystallite size, decoupling the two parameters and making them tunable *exogenously*.

The wettability of nanoporous materials such as metal–organic frameworks (MOFs) is of interest for a wide variety of applications, including but not limited to drug delivery^1−3^ and material separation,^4,5^ as well as gas^6−8^ and energy storage.^9^ MOFs are crystalline architectures composed of metal ions or clusters coordinated to organic linkers that assemble readily into complex structures containing stable cavities of different shapes and sizes.^10^

Among MOFs, there is a subclass, zeolitic imidazolate frameworks (ZIFs),^11^ that possess intriguing properties, such as flexibility and hydrophobicity, which make them particularly appealing for diverse applications. The effect of ZIF flexibility has already been investigated,^12−15^ with this property being utilized to intrude molecules that, according to traditional models, would not pass through the narrow pore apertures of the material. For example, in ZIF-8, the 6-membered ring (6 MR) apertures connecting the truncated octahedron cavities of the MOF have a diameter of 3.4 Å, which is very close to the van der Waals radius of water (3.2–3.6 Å),^16^ yet ZIF-8 is capable of stretching via gate-swinging of the imidazolate, allowing the MOF to be intruded by water when sufficient pressure is applied.^17^

Considering applications such as energy dissipation^18,19^ and mechanical,^20^ electrical^9,21^ or thermal energy storage,^22,23^ it is important to determine the heat flux that accompanies water intrusion-extrusion into-from the porous materials, and whether it affects the efficiency of the process and the stability of the material: information which is also important in other applications, such as gas storage. The heats of intrusion and extrusion of a liquid into and from a lyophobic porous material have been studied for a variety of porous materials by high pressure calorimetry.^20,24−26^ For ZIF-8, the overwhelming majority of these measurements have been performed at room temperature.^23,27−30^ With regard to potential applications, the effect of temperature is important, as the operating temperature is unlikely to be constant in real world applications, for example, in energy storage media.

In the majority of cases, the size of the cavities within porous materials is polydisperse, which can affect both the mechanical and the heat flux characteristics of porous materials. However, MOFs are peculiar because their cavities are a result of repeating units within the crystal structure of the solid. Thus, in principle, all the cavities of the porous medium are identical (the cavity size is monodisperse), with the exception of the unavoidable presence of defects.

In spite of this monodispersity, we recently discovered that in small (≤50 nm) crystallites (henceforth referred to as nanoZIF-8) the intrusion pressure is ∼5 MPa lower than in samples containing only larger crystal grains^31^ (denoted in the following as macroZIF-8). Remarkably, this significant difference in the intrusion pressure is not due to a change in the atomistic structure of the system, which remains essentially identical between nano- and macroZIF-8 (see figure S1 and table S1). Based on previous findings,^32^ Johnson et al.^31^ have shown that this reduction in intrusion pressure is due to a larger (external) surface area to volume ratio, *SA*/*V*, in smaller ZIF-8 crystallites. This larger *SA*/*V* corresponds to a larger fraction of ZIF-8 cages close to the surface of the crystallite. Water molecules inside these outermost cages are capable of forming hydrogen bonds with bulk water across the hexagonal windows, which eases the intrusion of these cages. Once these cages are filled, the next innermost cages are then in contact with already wetted cages. The lower intrusion pressure results in a reduction in the specific volume of water intruding the porous material. By simulation (presented herein), this corresponds to a reduction of the intruded water density of 6.5%, which is approximately 1 order of magnitude larger than the corresponding reduction in the density of bulk water over a decompression from 25 to 20 MPa (NIST data^33^). Importantly, part of the reduction of intrusion volume is due to *prewetting* of surface half-cavities,^31^ hence the genuine percentage reduction of confined water density is lower than this value. For the determination of the dependence of confined water on the crystallite size, which cannot be obtained from experiments, we resorted to atomistic simulations.

The dependence of the intrusion and extrusion pressures and the intrusion volume on the crystallite size immediately reveals the impact of this parameter on the mechanical energy associated with this process (*W* = *P* · Δ*V*). Comprehending the effect of crystallite size on other key intrusion/extrusion characteristics (within an operable temperature range) is thus paramount both for fundamental research and technological applications.

Therefore, following our results concerning the effect of crystallite size on the mechanical^31^ and structural^15^ parameters of water intrusion-extrusion, we have investigated whether crystallite size could be used to tune the heats of intrusion and extrusion, and indeed whether or not the trend with temperature reported by Lowe et al.^23^ for macroZIF-8 (commercial) would be similar for nanocrystallites of ZIF-8. The heats of intrusion/extrusion are of significant interest for potential thermal energy storage applications: on the one hand, the tuning of heat *exogenously* by crystallite size would be of great interest for tailoring systems to specific applications, whereas on the other hand, if the temperature of operating systems varies significantly, it is important to understand well how those temperature variations will affect performance.

Finally, we considered the ratio between the work done to/by the system (*W*) and the quantity of heat (*Q*) produced, and whether or not it was different for both the nano- and macroZIF-8 samples. This ratio is an important consideration when designing systems for specific applications: If *Q*/*W* > > 1, thermal energy dominates the system, which would be beneficial for thermal energy storage systems, while if *Q*/*W* < < 1, this would favor a mechanical system where very little overall heat would be exchanged with the environment.

The heats of intrusion and extrusion were measured by scanning transitiometry at various temperatures. Our results show that the absolute heat values for ZIF-8 were dependent on crystallite size (see figures S1, S6–S7, and table S1 for crystallite size analysis), as illustrated in Figure 1a, with the absolute heats of intrusion and extrusion (|*Q*_int/ext_|) for nanoZIF-8 consistently smaller than those of macroZIF-8. This relationship between nano- and macroZIF-8 was consistent across the temperature range.

**Figure 1:**
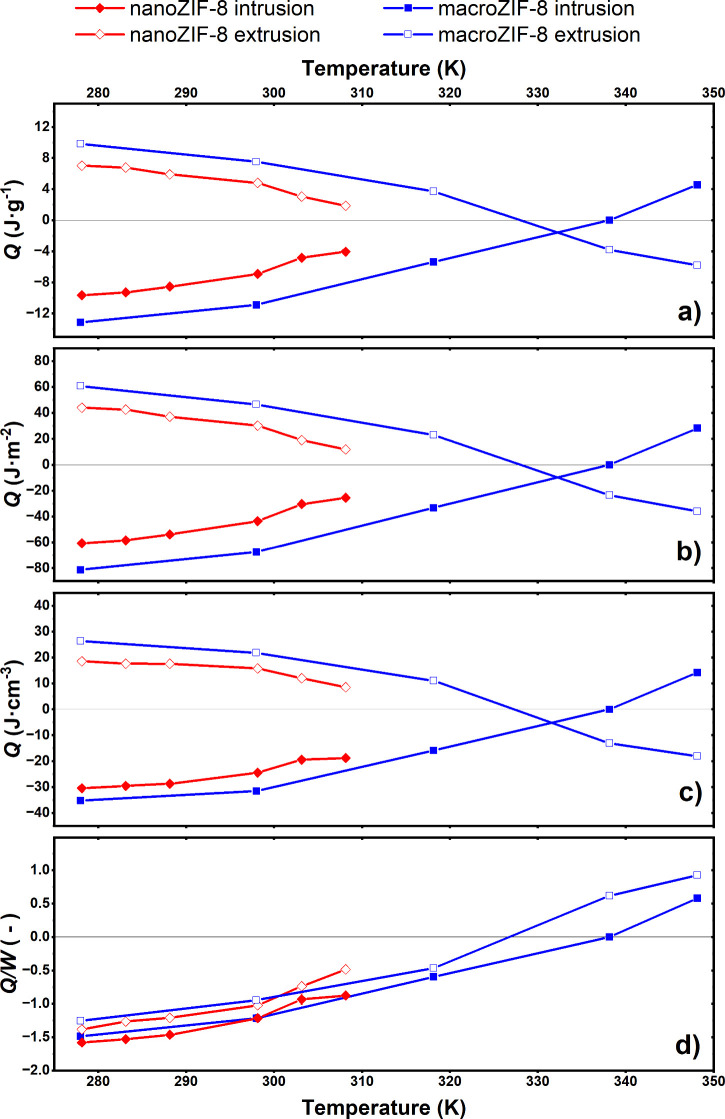
a) The heats of intrusion/extrusion vs temperature of nano- and macroZIF-8, including the surprising enthalpic inversion at higher temperature for macroZIF-8 (calorimetry signals reported in figures S2–S5 of the SI); b) the heats of intrusion/extrusion accounting for differences in BET surface area; c) the heats of intrusion/extrusion accounting for differences in intrusion volume; and d) the heat to work ratio for macro- and nanoZIF-8 at various temperatures. MacroZIF-8 heats of intrusion/extrusion data reproduced from Lowe et al.,^23^ with two additional extrusion points at high temperature reported in this work.

At 308.15 K, the nanoZIF-8 sample degraded, so no values were recorded at higher temperatures. Within this temperature range of 30 K, there was a clear trend of decreasing absolute heat with increasing temperature. For macroZIF-8, this strong, monotonic trend with temperature was similar and continued to higher temperatures. At 338.15 K, intrusion was athermic (*Q*_int_ ≃ 0) and extrusion was exothermic (*Q*_ext_ < 0). At 348.15 K, intrusion was endothermic (*Q*_int_ > 0) and extrusion was exothermic (*Q*_ext_ < 0): the signs of the heats of intrusion and extrusion were inverted at high temperature.

To understand whether the differences in heat measured were related to some inherent difference between the two samples, we normalized the heats of intrusion and extrusion at all temperatures by the BET surface area of the two materials. We considered this parameter because it could affect the size of the interface between the water and the ZIF-8 crystallites. We present these data in Figure 1b. We concluded from this that the surface area does not account for the difference between the two samples.

The intrusion volume of nanoZIF-8 was significantly lower than that of macroZIF-8 (figures S8–S9). Thus, in order to establish whether the difference in intrusion volume played a significant role or not, we normalized the heat values again, this time by the intrusion volume measured during the cycle. These results are presented in Figure 1c. There remained a significant difference between the nano- and macroZIF-8 samples.

We also noted that intrusion pressure (*P*_int_) (Figures 2, S7) was lower for nanoZIF-8 than for macroZIF-8, in good agreement with previous reports,^15,31^ and was also temperature dependent. For nanoZIF-8, intrusion and extrusion pressures increased with increasing temperature. Within the same temperature range, the trend for macroZIF-8 was similar to that of nanoZIF-8, but at very high temperature, the intrusion and extrusion pressures of macroZIF-8 decreased. This is a similar trend to that observed recently by Merchiori et al.^34,35^ for another hydrophobic porous MOF. They explained that this behavior was a consequence of the higher than expected vapor pressure within the porous matrix in conjunction with the reduction in the surface tension of water at the interface at higher temperatures.

**Figure 2:**
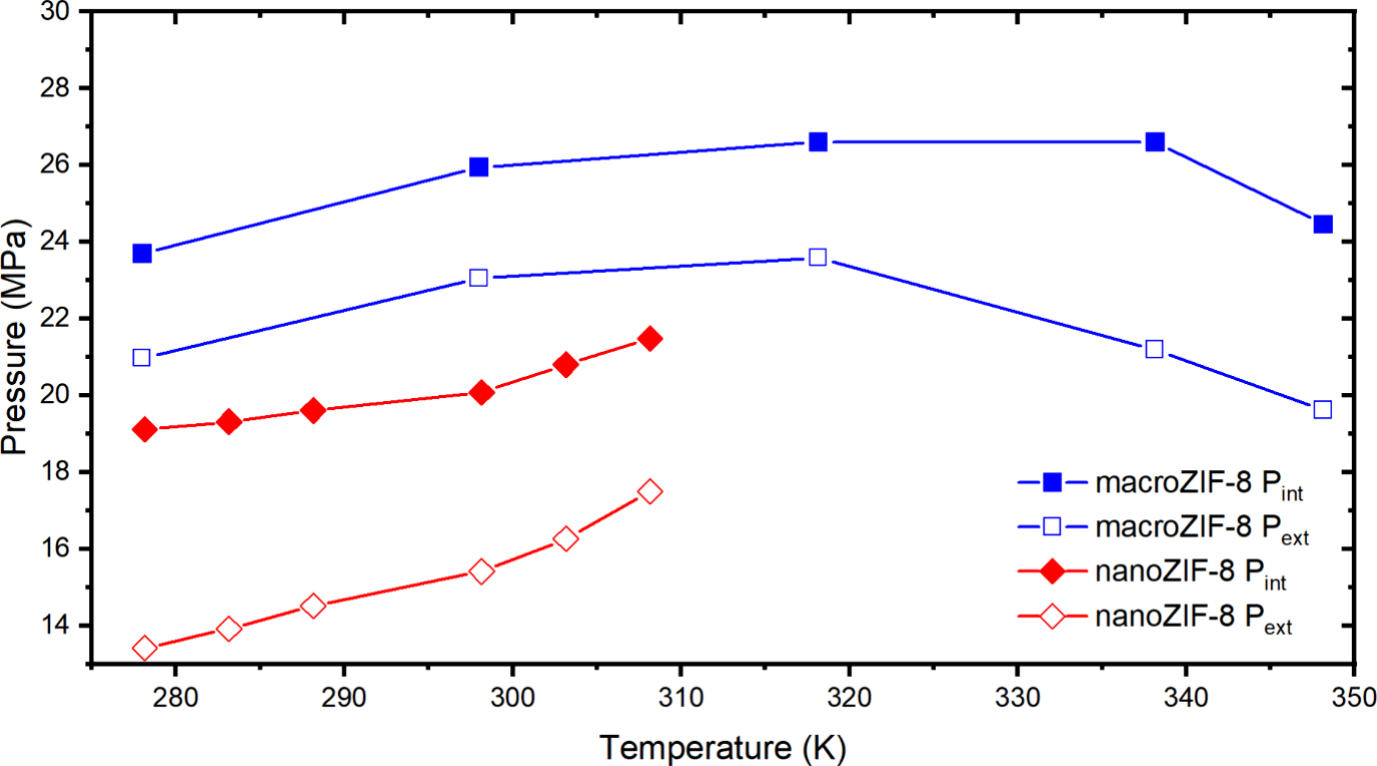
Intrusion (closed) and extrusion (open) pressures of macro- (blue) and nanoZIF-8 (red) at different temperatures.

Taking into account this relationship with intrusion/extrusion pressures and intrusion volume, we considered the mechanical work of intrusion and extrusion. This can be expressed as *W* = −*P* · Δ*V*, where *W* is the work done to the system, *P* is the intrusion/extrusion pressure, and Δ*V* is the specific volume intruded/extruded during the process. Since Δ*V* is negative for intrusion, *P* is positive, and exothermic heat has been established as a negative term, we have multiplied *P* · Δ*V* by −1 to make *W*_int_ positive and to clarify that the work of intrusion was energy put into the system.

Normalizing the heat of intrusion/extrusion by the work done to intrude/extrude the system accounts for not only the difference in intrusion/extrusion volumes, but also the difference in the intrusion/extrusion pressures. In Figure 1d, we present the heats of intrusion/extrusion for both nano- and macroZIF-8 normalized by work at each temperature. There was no significant difference between nano- and macroZIF-8 with respect to the ratio of heat to specific work (*Q*/*W*): i.e., the difference in crystallite size had *no effect* on the heat to work ratio of the system. This is surprising, as we already accounted for the difference in intrusion/extrusion volume in Figure 1c, suggesting that the pressure has a significant impact on the behavior of the system. We explore this further in the simulations described below.

In contrast, we saw a remarkable sensitivity of this heat/work ratio to the temperature of the experiment, suggesting that the contribution of mechanical and thermal energies in the wetting–dewetting process can be tuned by temperature. At low temperature, the system is dominated by heat effects (|*Q*/*W*| ≫ 1). This augmentation of the heat output would be very useful for thermal energy storage devices.

At higher temperatures, the system is dominated by mechanical effects (|*Q*/*W*| ≪ 1), which suits a mechanical system with low fluctuations in thermal energy. At ∼ 305 K, the thermal and mechanical energetic contributions are equivalent (|*Q*/*W*| ∼ 1), while above this temperature, the mechanical contribution is greater: a trend that is directly related to the heat of intrusion/extrusion values. At the highest measured temperatures, we saw that the heat contributions started to increase again, but with endothermic intrusion as discussed earlier. The fact that this trend of *Q*/*W* with temperature was independent of crystallite size is fascinating, as it decouples the tuning of the absolute heats of intrusion/extrusion from alterations to the heat to work balance of the system, i.e., the absolute heat of intrusion can be altered by crystallite size without changing the heat to work ratio, whereas a change of temperature would affect both characteristics.

The increase in the endothermic contributions for intrusion with increasing temperature demonstrated by the ZIF-8 + water system (Figure 1) is intriguing, and endothermic intrusion is of great interest in the field of energy, notably thermal energy conversion and storage: thermal energy is stored in the system upon intrusion and released upon extrusion. At 338.15 K, athermic intrusion was achieved, suggesting that at certain temperatures, the wetting of nanopores can be purely mechanical without an overall contribution to thermal energy.

To investigate the microscopic origin of the crystallite size effect on the heat of intrusion, atomistic simulations were performed. Direct atomistic simulations of crystallites of the relevant experimental sizes (>5 × 10^7^ atoms for a crystallite of ∼100 nm, plus the water in which it must be immersed) were unfeasible due to the computational power required. Moreover, our previous works^31,32^ (along with results presented in the Supporting Information) have demonstrated that the crystallite size affects the intrusion/extrusion characteristics as a consequence of *SA*/*V* rather than any changes to the structure of ZIF-8 (figure S1, table S1). Thus, we considered the same computational samples to compute the heat of intrusion for both nanoZIF-8 and macroZIF-8 using a two step approach.

In the first step, we performed free energy calculations (described in the Supporting Information) on a ZIF-8 slab immersed in water (figure S10). This enabled us to determine the number of water molecules in the equilibrium state of the confined liquid at 20 and 25 MPa, representing the H_2_O within the nano and macro crystallites’ experimental samples, respectively (Figure S11). In the second step, the heat of intrusion was computed on a triperiodic sample containing the number of water molecules determined during the first step, according to the procedure outlined in the subsequent paragraph.

The approach to compute the intrusion heat, presented in detail in a previous article,^23^ is discussed in the Supporting Information. Briefly, at constant pressure the heat of intrusion *Q*_int_(*P*) is equal to the variation of enthalpy Δ*H*_int_(*P*) of the process. In turn, the variation of enthalpy Δ*H*_int_(*P*) = Δ*U*_int_(*P*) + Δ(*PV*), where Δ*U*_int_(*P*) is the variation of internal energy, and *P* and *V* are the pressure and volume of the system. Δ*U*_int_(*P*) can be calculated as a difference of the (average) energy of the intruded and extruded states at a given pressure and temperature. To estimate the term Δ(*PV*), the intruded volume at the given pressure is required. This is obtained by computing the volume of bulk liquid corresponding to the water molecules intruded in the MOF at the relevant pressures, 20 MPa for nanoZIF-8 and 25 MPa for macroZIF-8. Note that the difference in the specific intrusion volume between nanoZIF-8 and macroZIF-8 contains an *apparent* term (see Johnson et al.^31^) whose effect must be disregarded. For this reason we have refrained from using experimental values of *V*_int_ for our analysis.

As mentioned before, for the second step we used a triperiodic sample containing the equilibrium amount of water, as opposed to the immersed slab used for the free energy calculations. This is because with the triperiodic sample we computed average quantities, namely the average energy necessary to compute Δ*U*_int_ with higher accuracy. In the slab sample, most water molecules remain in the bulk liquid, and significant energy fluctuations during constant-temperature simulations on this sample make the convergence of Δ*U*_int_ very slow.

We observed a 33% reduction of the absolute value of heat (intrusion became less exothermic at lower pressures), in line with the percentage reduction of *Q*_int_ between macro- and nanoZIF-8 measured experimentally (see Figure 1a, 36%).

Then, the origin of this pressure dependence of the *Q*_int_ was investigated.

Considering the relation *Q*_int_ = Δ*U*_int_(*P*) + Δ(*PV*), the effect of the crystallite size on the heat of intrusion *via P*_int_ is 2-fold. One is the direct effect, with pressure affecting the term Δ(*PV*). The other effect is indirect: at higher intrusion pressures the density of confined water is higher, which affects Δ*U*_int_. Distinguishing between the direct and indirect effect is impossible experimentally, but simulations allow us a greater flexibility to isolate the contributions. Both terms require the computation of the intruded volume at the two reference pressures, 20 and 25 MPa. Our free energy calculations (figure S11) have shown that at 20 MPa, ZIF-8 contains ∼37 water molecules per cage in its wet state, whereas at 25 MPa this value increases to ∼39. We remark that the change of *P*_int_ from nanoZIF-8 to macroZIF-8 caused a confined water density increase of 6.5%, more than 1 order of magnitude with respect to the increase of density of bulk water over the same pressure range, as obtained by NIST data.^33^

To more conveniently analyze the origin of the effect of the crystallite size on the heat of intrusion *via P*_int_, we wrote the variation of heat of intrusion Δ*Q* = *Q*_int_(25 MPa) – *Q*_int_(20 MPa) as follows:
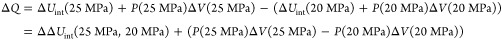
1where Δ*U*_int_(*P*) is the variation of internal energy during intrusion at pressures of 25 and 20 MPa, ΔΔ*U*_int_(25 MPa, 20 MPa) is the change of variation of internal energy of intrusion due to the change of intrusion pressure, and Δ*V*(*P*) is the volume of intrusion, which is dependent on pressure, as we have shown. The method by which we obtained Δ*U*_int_(*P*) from atomistic simulations is discussed in the Supporting Information. Our analysis revealed that ∼87% of Δ*Q* arose from the internal energy contribution ΔΔ*U*_int_(25 MPa, 20 MPa), while only ∼13% was attributed to the mechanical part. Indeed, this was not surprising considering the large change of density of filling upon the increase of pressure from 20 to 25 MPa.

To summarize, the reduction of intrusion heat obtained from atomistic simulations at 300 K (∼33%) was consistent with the reduction in experimental heat measurements of macro- vs nanoZIF8 (∼36%). Our simulations showed that this was mostly due to the reduction of the degree of filling at lower pressures, mostly affecting the variation of internal energy upon intrusion.

We have demonstrated that the heats of intrusion and extrusion are tunable both by the crystallite size of the MOF and also by the operating temperature of the intrusion-extrusion process. We have shown that the absolute heats of intrusion and extrusion for nanoZIF-8 were lower than those of macroZIF-8 at all measured temperatures. Remarkably, the ratio between thermal and mechanical energies was *independent* of the crystallite size within a window of 30 K, while being sensitive to temperature, allowing for the tuning of the absolute values of heat by crystallite size without altering the heat to work ratio. Future work will focus on a greater understanding of the mechanisms behind these phenomena and the potential expansion of this dependence with other ZIFs.

## Experimental Section

### MacroZIF-8

The sample with the largest size, referred to as macroZIF-8, was acquired from Merck as Basolite Z1200, CAS#59061-53-9, Lot #STBG5528 V.

### NanoZIF-8 Synthesis

The synthesis of the small sample, referred to as nanoZIF-8 was performed according to a protocol already reported in the literature,^36^ which is further detailed below.

Zn(NO_3_)_2_·6H_2_O, 2-methylimidazole and methanol were acquired from Sigma-Aldrich and used as received. To prepare a batch of ZIF-8 nanocrystallites, two methanolic precursor solutions of the metal and the ligand were prepared in two different Erlenmeyer flasks: the precursor solution of the metal was prepared by dissolving Zn(NO_3_)_2_·6H_2_O 1.467 g in 100 mL methanol (0.049 M); the precursor solution of the ligand was prepared by dissolving 2-methylimidazole 3.245 g in 100 mL methanol (0.395 M). The two solutions were separately mixed until complete dissolution of both components had been achieved. The first solution was then rapidly poured into the second one, while the latter was stirred continuously. The obtained solution was vigorously stirred for a further 3 min for sample nanoZIF-8a, 5 min for sample nanoZIF-8b, and 15 min for sample nanoZIF-8c until the solution became cloudy. The cloudy suspension was immediately decanted into four different 50 mL Falcon vials and centrifuged at 150 Hz for 30 min. For each vial, the supernatant solution was disposed of, and the precipitated product pellet was washed twice with fresh methanol (first with 60 mL and then with 30 mL) and centrifuged after each wash at 150 Hz for 60 min. After being centrifuged once more, the pellet product was left to dry at room temperature and then finely crushed with a mortar to get a homogeneous white powder. Analysis of the samples informed us that they were all identical, so they were combined into a homogeneous sample.

### Calorimetry Measurements

A BRG-tech Scanning Transitiometer, located at the University of Silesia in Katowice,^23−25,30^ was used to simultaneously record the pressure–volume isotherms and thermograms of the liquid intrusion/extrusion of both nanoZIF and macroZIF-8. In these experiments, two calorimeter cells, one sample, and one reference, each rated for a maximum pressure of 200 MPa, were attached to a manifold that is connected to a single high-pressure line leading to a high-pressure piston and stepper motor rated for a maximum pressure of 700 MPa. In this arrangement, both cells experience the same applied pressure generated by the pump.

For each sample, a mass of ZIF-8 was weighed into a small stainless steel capsule with an outer diameter of 6 mm and an inner wall thickness of 0.5 mm. The bottom end of the tube was plugged with a ball of filter paper, compressed at one end, and then weighed. The steel capsule was filled with ZIF-8 sample material and weighed again. The difference between both empty and full capsule provided the mass of ZIF-8 in the capsule. A final filter paper plug was placed within the tube to keep the powder held in place. The capsule was transferred into the measuring cell, where it rests on a spring holding the capsule in place. Then, both calorimeter cells were filled with distilled and degassed water, taking care to not add air bubbles into the cells. The change in volume is calculated from the number of steps needed to push the volume of water through the high pressure line by the stepper motor.

### TEM Measurements

TEM measurements were carried out by using a FEI Tecnai F20 (FEI Co., Hillsboro, OR, United States) electron microscope operated at 200 keV equipped with an X-ray Dispersive Energy (EDX) spectrometer. Several milligrams of ZIF-8 powder were dispersed in ethanol and sonicated to obtain a homogeneous dispersion. A drop of the dispersion was dropped onto the sample holder and inserted into the TEM column for observation. For each sample, various areas were examined. Example TEM images are included in figure S6 and the histograms of the size distribution from the TEM images are presented in figure S7 of the Supporting Information.

### X-ray Diffraction Measurements

A Bruker D8 Discover X-ray diffractometer was used with a LYNXEYE-XE detector using CuKα1 radiation (*λ* = 1.5418 Å) and Bragg–Brentano θ:2θ geometry. The data collection was conducted at room temperature, between 5° and 80° with a step of 0.02° and a dwell time of 2 s per step. Crystallite size (coherent domain size) was estimated using the LeBail^37^ method implemented in Fullprof Suite.^38^ ZIF-8 patterns were generated assuming ideal cubic symmetry with space group I −43m (#217) and laue class m-3m. The diffraction peaks were visibly broadened in comparison to the standard pattern of Al_2_O_3_, which was used to measure the machine resolution function. The quantitative analysis of the size broadening was achieved using the spherical harmonics formalism defined by Järvinen.^39^ The refined patterns and the data are presented in figure S1 and table S1 respectively.

## Supporting Information Available

Further details on the computational methods used during this investigation; XRD diffractograms of macroZIF-8, nanoZIF-8, and the simulated pattern; structure of ZIF-8, rendered from the CIF file in the VESTA software; Table S1, microstructural parameters refined using the LeBail method; plots of compression of macroZIF-8 with the pressure, volume, and heat flux overlaid and plotted against time; plots of decompression of macroZIF-8, compression of nanoZIF-8, and decompression of nanoZIF-8 with the pressure, volume, and heat flux overlaid and plotted against time; TEM images of nanoZIF-8 and macroZIF-8; histograms of the crystallite size distribution of both macro- and nanoZIF-8; PV isotherms of macro- and nanoZIF-8 at different temperatures; intrusion volumes vs temperature for both macro- and nanoZIF-8; image of the slab of ZIF-8 not yet fully intruded, used to calculate the difference in intrusion volume by RMD simulations at 20 and 25 MPa; and free energy plots for the filling of ZIF-8 cages at 300 K vs number of water molecules (PDF)
Transparent Peer Review report available (PDF)
