# Supplementary material for: Crystallite Size Effects on the Heat of Water Intrusion/Extrusion into/from Metal–Organic Frameworks
Source: J Phys Chem Lett. 2025 Feb 20;16(8):2089–96. doi: 10.1021/acs.jpclett.4c02639 (PMC11873925; doi:10.1021/acs.jpclett.4c02639)

Crystallite Size Effects on the Heat of Water Intrusion/Extrusion into/from Metal-organic Frameworks

Liam J. W. Johnson,[†,‡,⊥] Alexander R. Lowe,[¶,⊥] Andrea Le Donne,[§,⊥] Emre Arkan,[¶] Sebastiano Merchiori,[§] Luis Bartolomé,[†] Eder Amayuelas,[†] Diego Mirani,[‖] Gabriel A. López,[‡] Giulia Grancini,[‖] Mirosław Chorążewski,[*,¶] Simone Meloni,[*,§] and Yaroslav Grosu[*,†,¶]

†*Centre for Cooperative Research on Alternative Energies (CIC energiGUNE), Basque Research and Technology Alliance (BRTA), Vitoria-Gasteiz, 01510, Spain*

‡*Department of Physics, Faculty of Science and Technology, University of the Basque Country (UPV/EHU), Barrio Sarriena s/n, Bilbao 48490 Leioa, Spain*

¶*Institute of Chemistry, University of Silesia in Katowice, Szkolna 9, 40-006 Katowice, Poland*

§*Dipartimento di Scienze Chimiche e Farmaceutiche (DipSCF), Università degli Studi di Ferrara (Unife), Via Luigi Borsari 46, I-44121, Ferrara, Italy*

‖*Department of Chemistry and INSTM University of Pavia Via Taramelli 14, Pavia I-27100, Italy*

⊥*These authors contributed equally*

E-mail: miroslaw.chorazewski@us.edu.pl; simone.meloni@unife.it; ygrosu@cicenergigune.com

Phone: +34 945 29 71 08

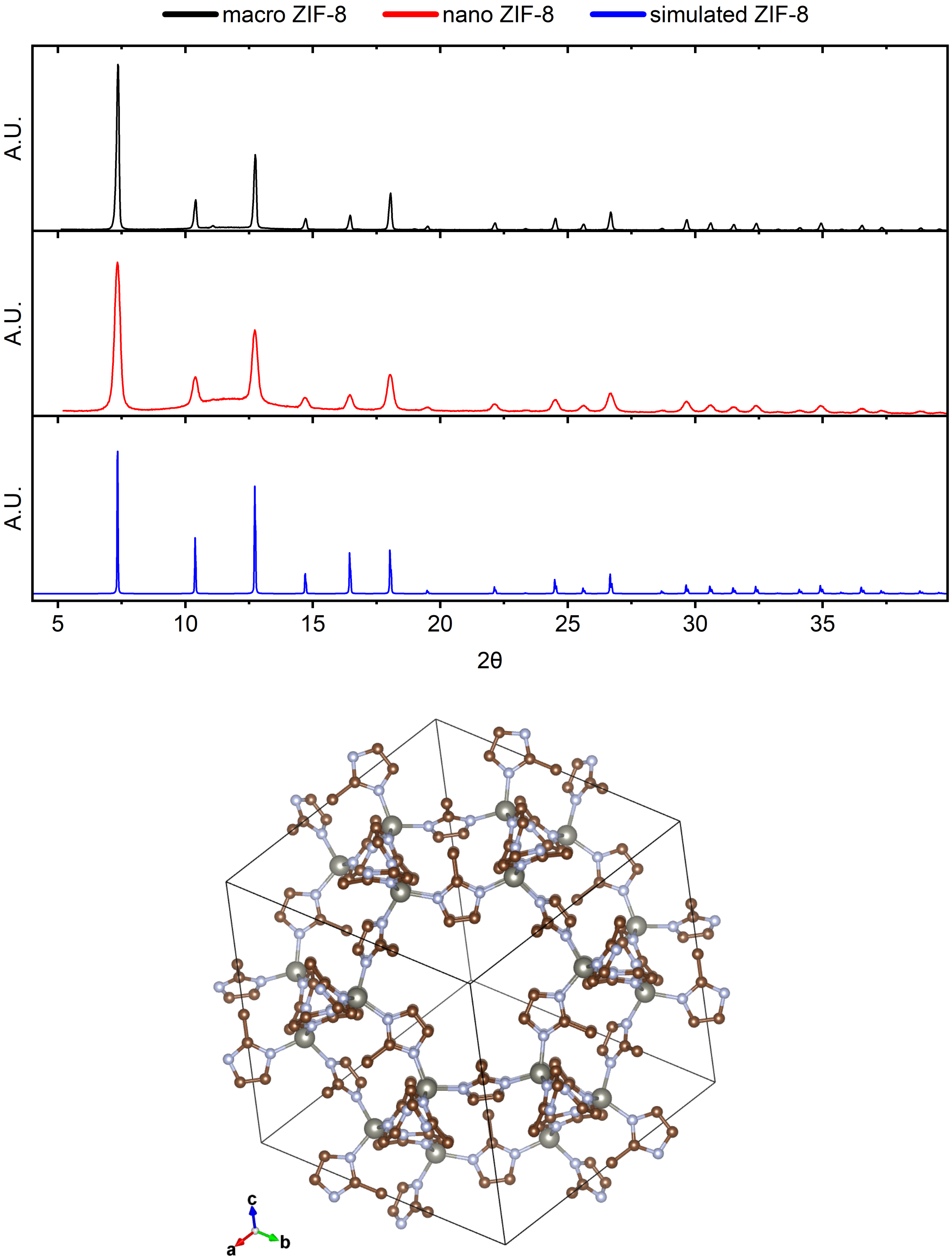

Figure S1: XRD patterns of macro ZIF-8 (black), nano ZIF-8 (red), and the simulated pattern from the CIF file.[1] The small bump around 10-15° is an amorphous contribution either from the grease used during sample preparation or some disorder within the ZIF-8 structure.

Table S1: The microstructural parameters refined using the LeBail method.[2] The average size is given in Ångströms alongside the anisotropy (contained within the braces), as well as the average strain and its associated anisotropy {}.

Sample ID	Lattice Parameter (Å)	Av. Size {anis.} (Å)	Strain {anis.} (%)
Macro	17.03	1947.00 {0}	20.52 {3}
Nano	17.02	406.44 {5}	33.46 {8}

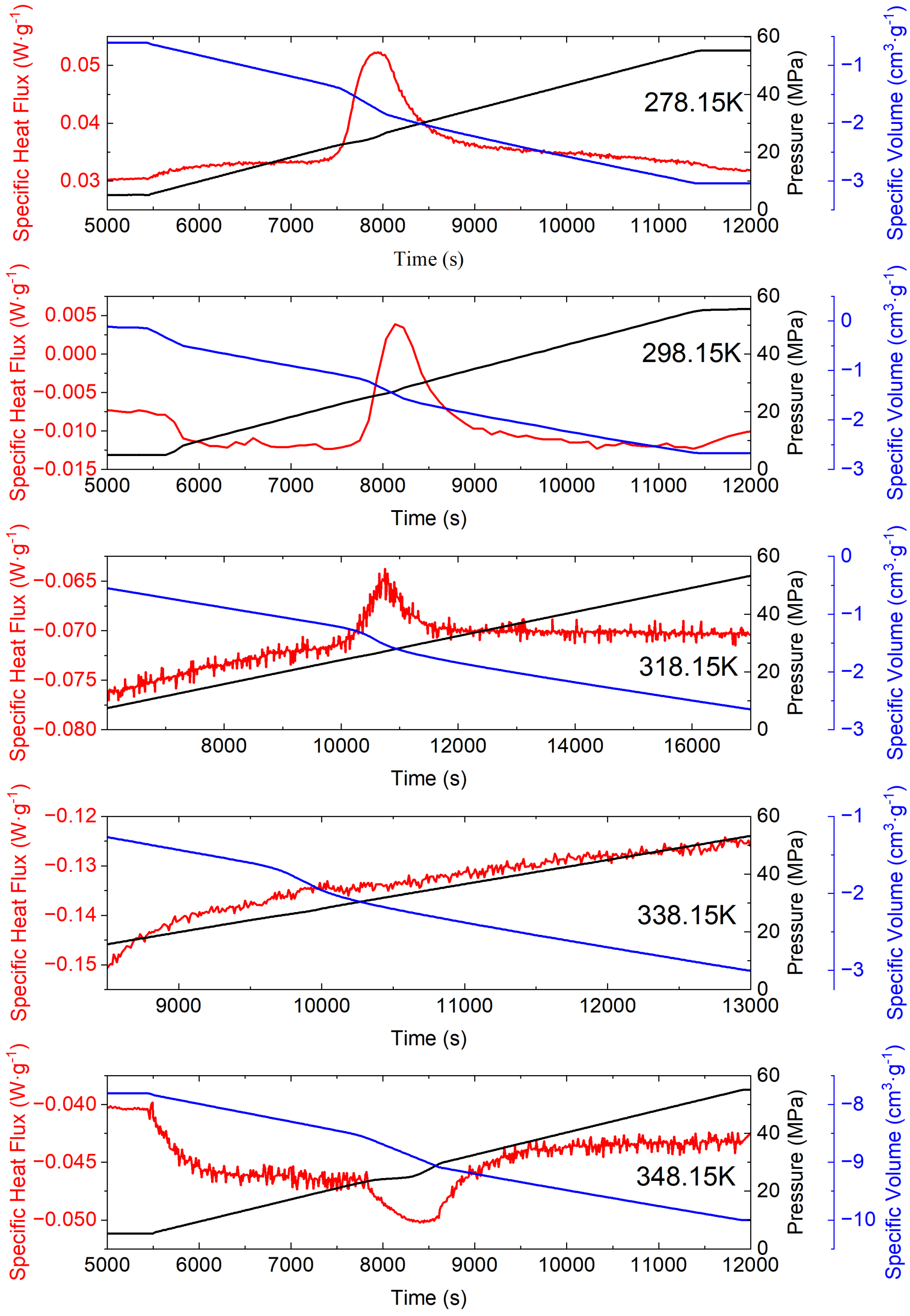

Figure S2: Compression heats of macroZIF-8 measured in the transitiometer experiments at different temperatures (red) overlaid with increasing pressure (black), and the change in volume (blue).

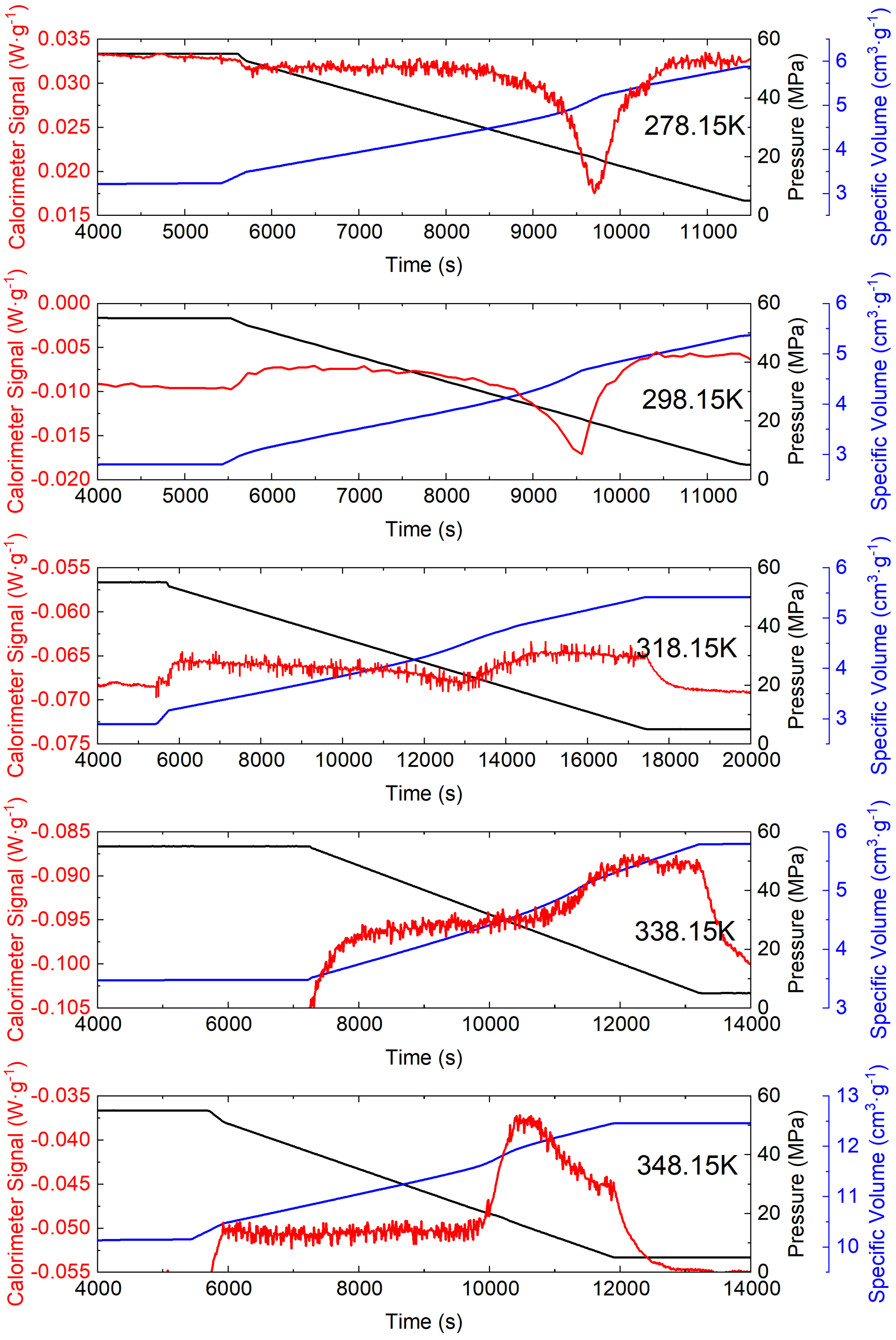

Figure S3: Decompression heats of macroZIF-8 measured in the transitiometer experiments at different temperatures (red) overlaid with decreasing pressure (black), and the change in volume (blue).

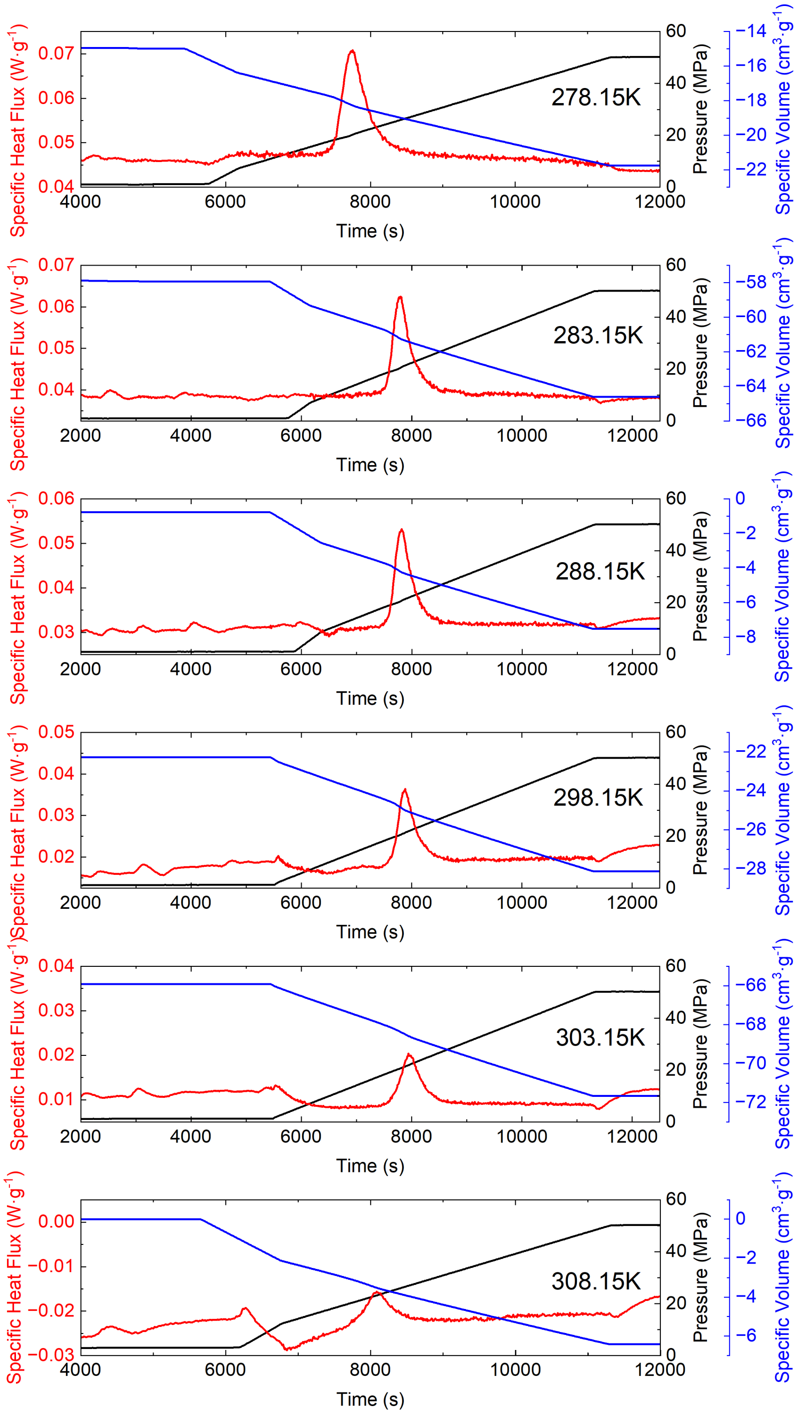

Figure S4: Compression heats measured in the transitiometer of nanoZIF-8 at different temperatures (red) overlaid with increasing pressure (black), and the change in volume (blue).

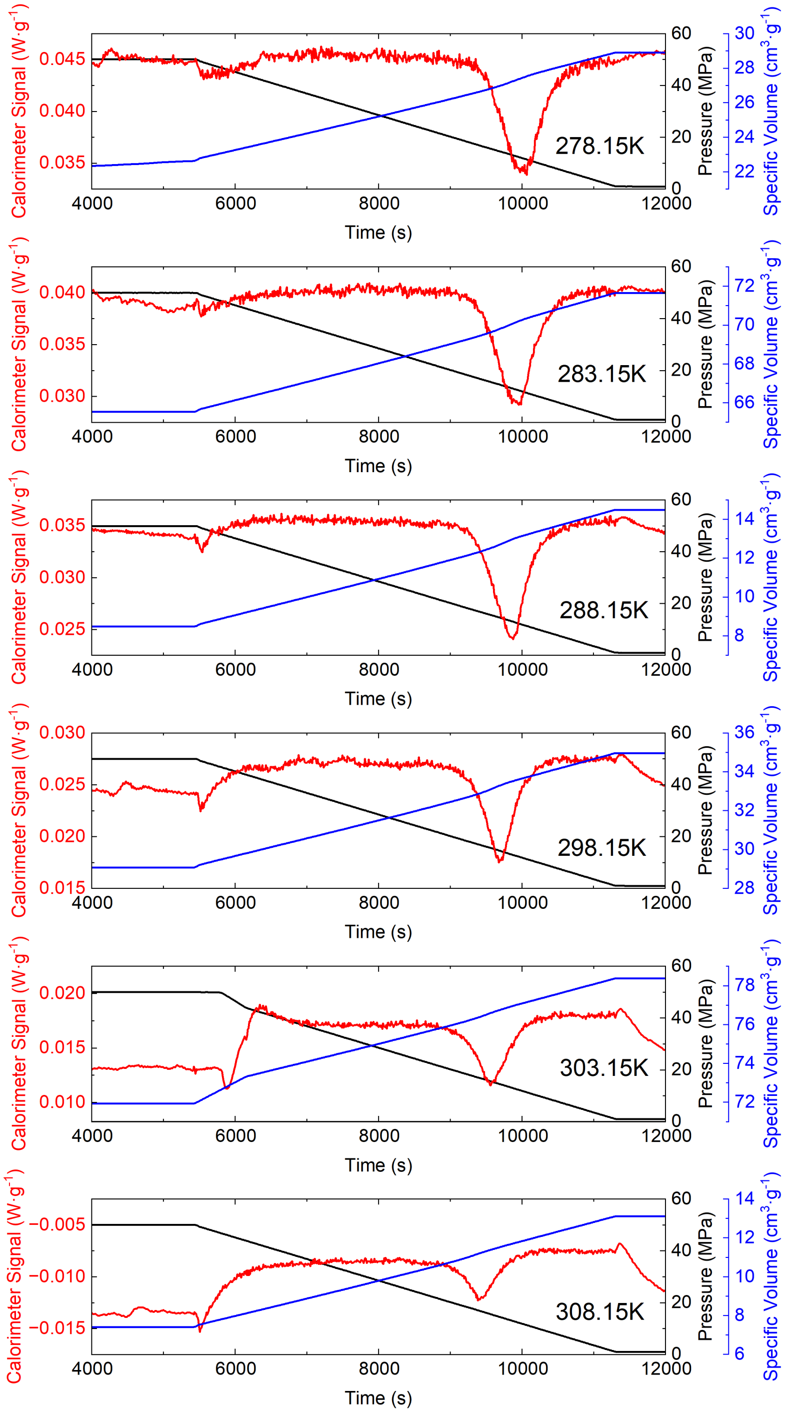

Figure S5: Decompression heats measured in the transitiometer of nanoZIF-8 at different temperatures (red) overlaid with decreasing pressure (black), and the change in volume (blue).

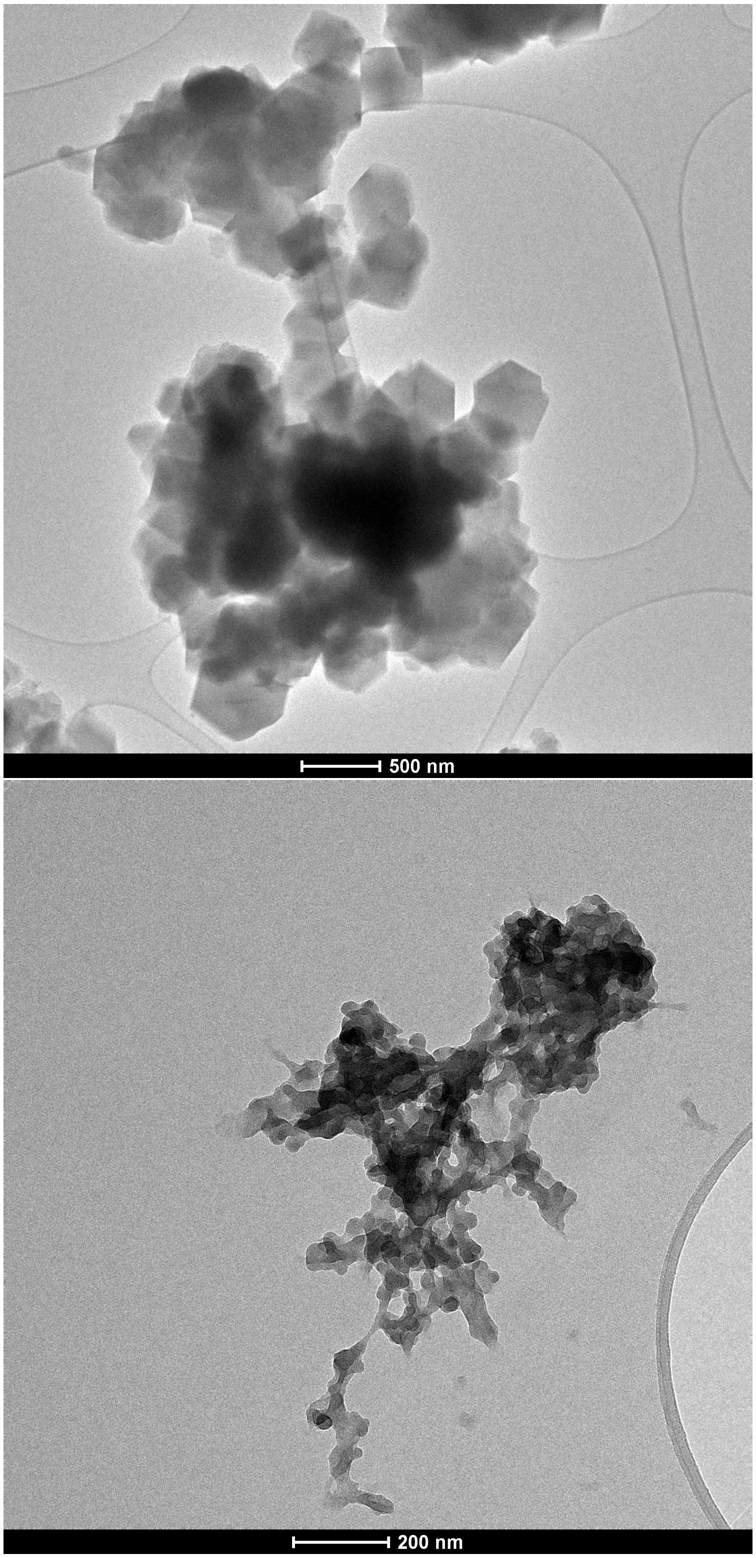

Figure S6: TEM images of macro- (above) and nanoZIF-8 (below)

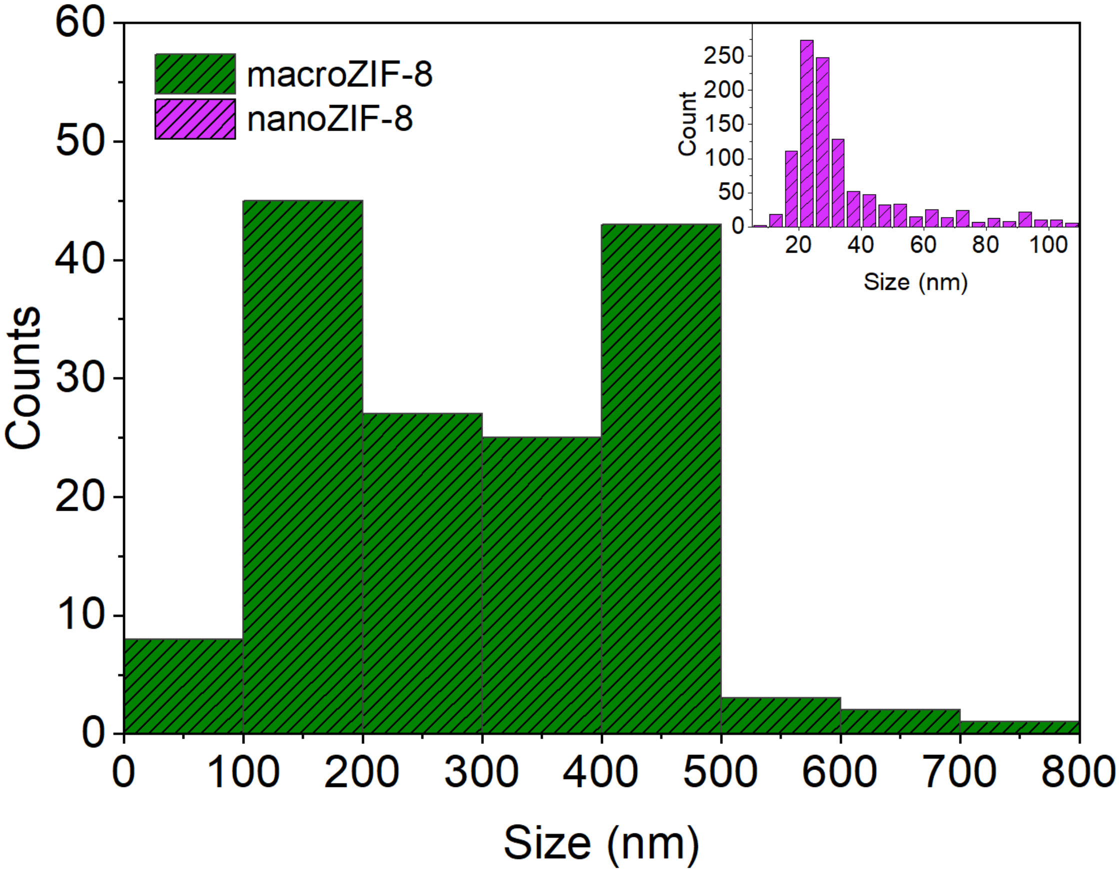

Figure S7: TEM histograms of particle size for the two ZIF-8 samples measured in this work.

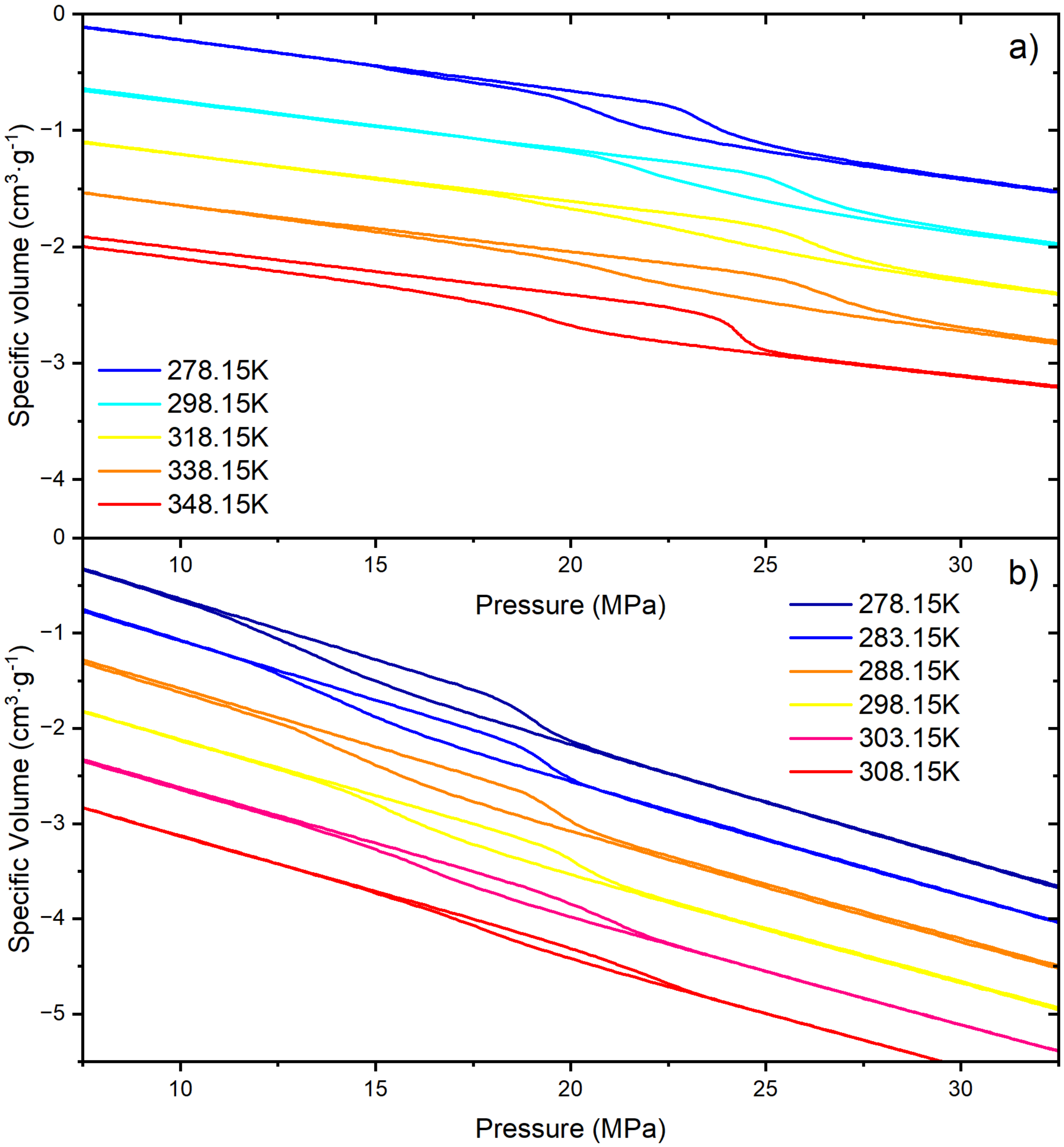

Figure S8: a) PV isotherms measured in the transitiometer for the macroZIF-8 + water system from 278.15 K up to 348.15 K and b) PV isotherms measured in the transitiometer for the nanoZIF-8 + water system from 278.15 K up to 308.15 K.

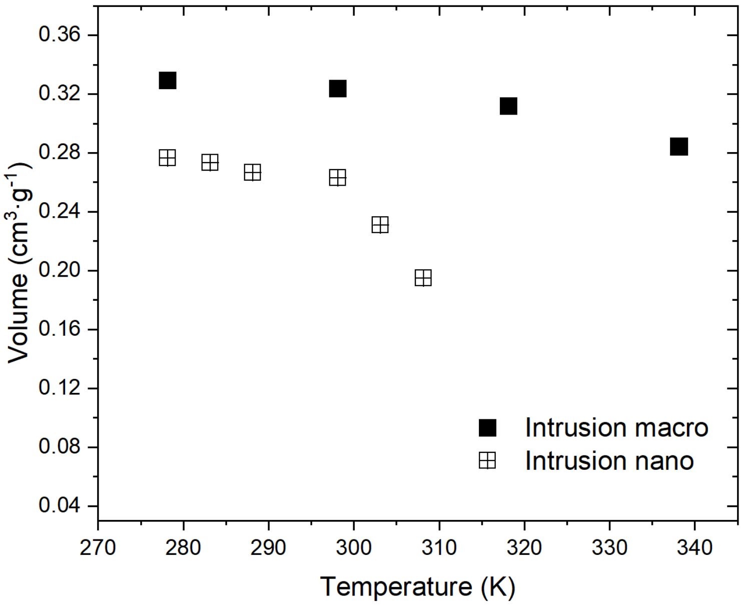

Figure S9: Intrusion volumes of macro- (closed squares) and nanoZIF-8 (open squares) at different temperatures.

Computational Methods

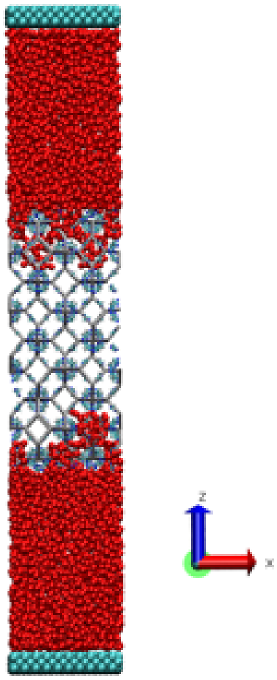

Figure S10: Image of the slab of ZIF-8, not yet fully intruded. The ZIF-8 slab was used to calculate the difference of intrusion volume *via* restrained molecular dynamics simulations at 20 and 25 MPa.

To explain the experimental variation of the heat of intrusion over ZIF-8 particle size, we used molecular dynamics simulations to compute the heat generated during the intrusion process. At constant pressure, the heat flow is equal to the enthalpy variation of the system (ΔH), so, to calculate the ΔH, simulations were used to compute the ensemble average value of H.

$$Q = \Delta H = \Delta U + P\Delta V \tag{1}$$

The variation of internal energy (ΔU) and the variation of volume (ΔV, multiplied by the constant pressure) were calculated along the simulations when the system was completely intruded and completely extruded. We used a tri-periodic $2 \times 2 \times 2$ ZIF-8 supercell computational sample, bulk water (bulk water together with ZIF-8 supercell represents the extruded state) and a fully intruded $2 \times 2 \times 2$ ZIF-8 supercell. The LAMMPS package[3] was employed

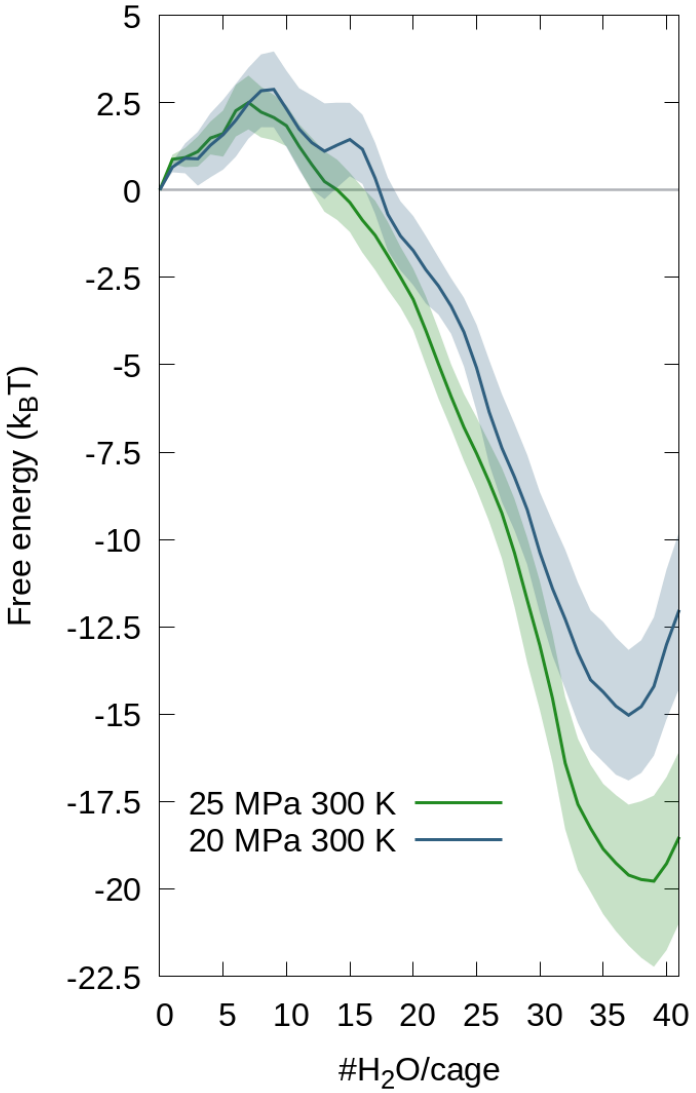

Figure S11: Reproduced from [3]. Copyright 2023 American Chemical Society. Free energy of filling, expressed in k_BT, against the number of water molecules inside a ZIF-8 cage at 20 (blue) and 25 MPa (green). Reducing the pressure, the energy of the stable-state is reduced by $\sim 5\ k_BT$. Moreover, the minimum is also reached with fewer water molecules, moving from 39 to 37 molecules per cage.

by applying the NPT ensemble (the number of particles, pressure and temperature were constant). The temperature was set to 300 K by applying a Nosè-Hoover chain thermostat with a coupling constant of 0.1 ps.

In previous work, the effect of ZIF-8 particle size on intrusion pressure was been studied: nanoZIF-8 presented a lower intrusion pressure (20 MPa) with respect to the larger commercial sample (macro ZIF-8, 25 MPa). This was rationalised by the greater surface area to volume ratio, which increases the number of cages in contacted with already wetted cages (as the surface half-cages are already wetted), stabilising the intruded state *via* cross-cage hydrogen bonding, and thus lowering the intrusion pressure.[4,5] The reduction of intrusion pressure also affects the number of water molecules inside the ZIF-8 cages. Applying restrained molecular dynamics for a ZIF-8 slab sample (figure S10),[6] the energy profile along the intrusion process has been calculated, revealing two wetted stable states (free energy minima) with different numbers of water molecules inside a single ZIF-8 cage (see figure S11, reproduced from Johnson et al.[5] Copyright 2023 American Chemical Society).

In order to consider this reduction in intrusion volume, the filled state was simulated at two different levels of filling: 39 (25 MPa) and 37 (20 MPa) molecules per cage to represent macro- and nanoZIF-8 respectively. The pressure was controlled by the Tobias-Klein-Martyna barostat[7] with a coupling constant of 1 ps. The force field of Zheng et al.[8] was used to model the potential energy of ZIF-8 combined with the TIP4P/2005 model of water, a setup already successfully applied in previous publications.[4,6] The duration of the simulations for each system was 80 ns, which was divided into 10 ns of thermalisation and 70 ns of production run.

Acknowledgement

This project has received funding from the European Union's Horizon 2020 research and innovation programme under grant agreement No. 101017858. We also acknowledge the support of the Basque Goverment through the IT-1714-22 project. This work is also part of the grant RYC2021-032445-I funded by MICIN/AEI/10.13039/501100011033 and by the European Union NextGenerationEU/PRTR and also received financial support based on Decision No. 2021/43/D/ST5/00062 from the National Science Center (Poland). S. Meloni acknowledges PRACE for computational access to Marconi 100 at CINECA (Bologna, Italy). A. Le Donne, S. Merchiori, and S. Meloni acknowledge the computational support of CINECA through grant IscrB LAUREATE (HP10B2KZAX). A.R. Lowe and M. Chorążewski are grateful for the financial support based on Decision No. 2018/31/B/ST8/00599 from the National Science Centre (Poland). We also thank the University of Silesia Machine Shop for helping maintain the Scanning Transitiometer. L.J.W. Johnson is grateful to Francisco Bonilla for their assistance with TEM measurements.

References

(1) Fairen-Jimenez, D.; Moggach, S. A.; Wharmby, M. T.; Wright, P. A.; Parsons, S.; Düren, T. Opening the Gate: Framework Flexibility in ZIF-8 Explored by Experiments and Simulations. *Journal of the American Chemical Society* **2011**, *133*, 8900–8902.

(2) Le Bail, A.; Duroy, H.; Fourquet, J. L. Ab-initio structure determination of LiSbWO6 by X-ray powder diffraction. *Materials Research Bulletin* **1988**, *23*, 447–452.

(3) Thompson, A. P.; Aktulga, H. M.; Berger, R.; Bolintineanu, D. S.; Brown, W. M.; Crozier, P. S.; in 't Veld, P. J.; Kohlmeyer, A.; Moore, S. G.; Nguyen, T. D.; Shan, R.; Stevens, M. J.; Tranchida, J.; Trott, C.; Plimpton, S. J. LAMMPS - a flexible simulation tool for particle-based materials modeling at the atomic, meso, and continuum scales. *Computer Physics Communications* **2022**, *271*, 108171.

(4) Johnson, L. J. W.; Paulo, G.; Bartolomé, L.; Amayuelas, E.; Gubbiotti, A.; Mirani, D.; Le Donne, A.; López, G. A.; Grancini, G.; Zajdel, P.; Meloni, S.; Giacomello, A.; Grosu, Y. Optimization of the Wetting-Drying Characteristics of Hydrophobic Metal Organic Frameworks via Crystallite Size: The Role of Hydrogen Bonding between Intruded and Bulk Liquid. *Journal of Colloid and Interface Science* **2023**, *645*, 775–783.

(5) Johnson, L. J. W.; Mirani, D.; Le Donne, A.; Bartolomé, L.; Amayuelas, E.; López, G. A.; Grancini, G.; Carter, M.; Yakovenko, A. A.; Trump, B. A., et al. Effect of Crystallite Size on the Flexibility and Negative Compressibility of Hydrophobic Metal–Organic Frameworks. *Nano Letters* **2023**, *23*, 10682–10686.

(6) Tortora, M.; Zajdel, P.; Lowe, A. R.; Chorażewski, M.; Leão, J. B.; Jensen, G. V.; Bleuel, M.; Giacomello, A.; Casciola, C. M.; Meloni, S.; Grosu, Y. Giant Negative Compressibility by Liquid Intrusion into Superhydrophobic Flexible Nanoporous Frameworks. *Nano Letters* **2021**, *21*, 2848–2853.

(7) Martyna, G. J.; Tobias, D. J.; Klein, M. L. Constant pressure molecular dynamics algorithms. *The Journal of chemical physics* **1994**, *101*, 4177–4189.

(8) Zheng, B.; Sant, M.; Demontis, P.; Suffritti, G. B. Force field for molecular dynamics computations in flexible ZIF-8 framework. *Journal of Physical Chemistry C* **2012**, *116*, 933–938.

TOC Graphic

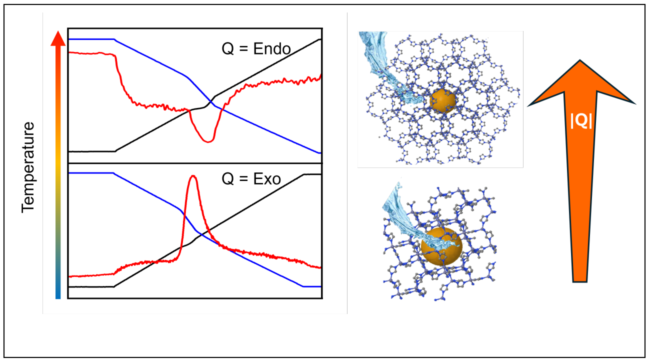

Supplement: Supplementary file 1 — jz4c02639_si_001.pdf [file jz4c02639_si_001.pdf]
