# Supplementary material for: Crystallite Size Effects on the Heat of Water Intrusion/Extrusion into/from Metal–Organic Frameworks
Source: J Phys Chem Lett. 2025 Feb 20;16(8):2089–96. doi: 10.1021/acs.jpclett.4c02639 (PMC11873925; doi:10.1021/acs.jpclett.4c02639)
Supplement: Supplementary file 2 — jz4c02639_si_002.pdf [file jz4c02639_si_002.pdf]

jz-2024-02639p.R1

Name: Peer Review Information for "Crystallite Size Effects on the Heat of Water Intrusion/Extrusion into/from Metal-organic Frameworks"

First Round of Reviewer Comments

Reviewer: 1

Comments to the Author

This study investigates the effect of crystallite size and temperature on the heats of water intrusion and extrusion in ZIF-8. The authors compare macroZIF-8 and nanoZIF-8 samples across various temperatures, measuring intrusion/extrusion pressures, volumes, and associated heats. The research presents the following novel findings, including:

NanoZIF-8 exhibits lower absolute heats of intrusion/extrusion compared to macroZIF-8 across all temperatures.

The heat-to-work ratio (Q/W) is independent of crystallite size but sensitive to temperature.

Atomistic simulations suggest that the crystallite size effect on intrusion heat is primarily due to differences in intrusion pressure and subsequent changes in cavity filling.

The findings have direct implications for potential applications of ZIF-8 alike materials in energy storage, dissipation, and thermal management systems. The ability to tune the thermal and mechanical energy contributions by adjusting temperature and crystallite size offers new possibilities for tailoring these materials for specific energy-related applications.

However, several issues warrant consideration:

a) The paper occasionally uses informal phrasing, such as "curious." While this does not significantly impair understanding, addressing such instances would enhance the overall polish of the manuscript.

b) Insufficient detail is provided regarding the two simulation models (nano and macro ZIF-8). Specifically, the paper lacks information on how nano and macro ZIF-8 structures were constructed and how the ZIF-8 surface layer was built and terminated. The current information only states that atomistic simulations considered two pressure points (20 and 25 MPa) to mimic water intrusion in nano- and macroZIF-8. Justification for selecting these specific pressures is absent.

c) The paper omits crucial details about water adsorption in nano and macro ZIF-8, such as hydrogen bond networks and adsorption sites, along with the associated insights these details might provide.

In light of these issues, I recommend a major revision for this manuscript to address these gaps in methodology and analysis.

Reviewer: 2

Comments to the Author

In this manuscript, the heats of intrusion-extrusion into/from ZIF-8 + water systems of various ZIF-8crystallite sizes were measured at different temperatures. Their results show that the absolute heat values for water intrusion-extrusion into/out of ZIF-8 are dependent on crystallite size. However, the heat/work ratio of the intrusion-extrusion processes was dependent on temperature whilst independent of crystallite size. The results reported here are interesting and important. However, I have several comments and suggestions outlined below, which should be addressed.

1. P6 L22-23: "At 338.15 K, intrusion was athermic ($Q_{int} \simeq 0$) and extrusion was exothermic ($Q_{ext} < 0$)." I cannot understand why does the extrusion have quite different heat effect with its reverse process, intrusion?

2. P7 L14-15: "at very high temperature, the intrusion and extrusion pressures of macroZIF-8 decreased". More discussion should be given to explain the underlying mechanism.

3. Detailed pore structures of two ZIF-8 samples should be provided.

4. Are there any differences in chemical composition of two ZIF-8 samples?

5. Where is the difference in particle size from? TEM images of two samples should be given to illustrate the difference in particle size.

6. In Abstract, it claims that These results were mirrored in atomistic simulations, where the reduction of intrusion heat by reducing crystallite size was comparable to the values obtained experimentally. Simulation details should be given to describe how they define the particle sizes of two samples.

Author's Response to Peer Review Comments:

Please see the attached PDF file for our response to the reviewers' comments.

Reviewer: 1

Recommendation: This paper is probably publishable, but major revision is needed; I do not need to see future revisions.

Comments:

This study investigates the effect of crystallite size and temperature on the heats of water intrusion and extrusion in ZIF-8. The authors compare macroZIF-8 and nanoZIF-8 samples across various temperatures, measuring intrusion/extrusion pressures, volumes, and associated heats. The research presents the following novel findings, including:

NanoZIF-8 exhibits lower absolute heats of intrusion/extrusion compared to macroZIF-8 across all temperatures.

The heat-to-work ratio (Q/W) is independent of crystallite size but sensitive to temperature.

Atomistic simulations suggest that the crystallite size effect on intrusion heat is primarily due to differences in intrusion pressure and subsequent changes in cavity filling.

The findings have direct implications for potential applications of ZIF-8 alike materials in energy storage, dissipation, and thermal management systems. The ability to tune the thermal and mechanical energy contributions by adjusting temperature and crystallite size offers new possibilities for tailoring these materials for specific energy-related applications.

However, several issues warrant consideration:

a) The paper occasionally uses informal phrasing, such as "curious." While this does not significantly impair understanding, addressing such instances would enhance the overall polish of the manuscript.

We thank the reviewer for bringing this to our attention – in these instances, more suitable vocabulary has now been used.

b) Insufficient detail is provided regarding the two simulation models (nano and macro ZIF-8). Specifically, the paper lacks information on how nano and macro ZIF-8 structures were constructed and how the ZIF-8 surface layer was built and terminated. The current information only states that atomistic simulations considered two pressure points (20 and 25 MPa) to mimic water intrusion in nano- and macroZIF-8. Justification for selecting these specific pressures is absent.

We would like to thank the reviewer for drawing our attention to the lack of clarity on this point, confirmed by a corresponding question from reviewer two. For the sake of brevity imposed by a letter, in the original version of this manuscript the presentation of the modeling principles adopted in this

work was done mainly via *reference to two articles we published in 2023.*[1,2] *Your comments, along with those of reviewer two, convinced us that this was insufficient and in this amended version of the manuscript we discuss this in more detail.*

Considering the size of experimental crystallite, 35 nm for nanoZIF-8 and 100 nm or more for the macro counterpart, a brute force simulation approach, with the computational sample consisting of a crystallite of realistic size immersed in sufficient water would be computationally unfeasible. For this reason, we opted for an alternative approach based on the findings of two previous articles,[1,2] *where we showed that the intrusion/extrusion pressures grow with crystallite size despite no significant change in the structure of the MOF (figure S1 and table S1 of the Supporting Information of this amended version of the manuscript). In these articles, we showed that this relationship between intrusion pressure and ZIF-8 crystallite size is related to the external surface area to volume ratio, the latter of which is itself dependent upon crystallite size. This, together with i) the formation of hydrogen bonds between water molecules across hexagonal apertures of the ZIF-8 cavities that reduce the instability of the intruded state, and ii) the incomplete semi-cavities on the surface, which are exposed to the bulk liquid and are thus wet at atmospheric pressure, cause the intrusion pressure of nanoZIF-8 to be approximately 5 MPa lower than in the corresponding macroZIF-8 sample. In practice, the larger surface area/volume ratio reduces the intrusion free energy barrier, and thus nanoZIF-8 can be intruded at lower pressures.*

Based on the findings of these previous articles, summarized in the previous paragraph, here the size of the crystallite is not considered directly but rather by determining the heat of intrusion in a slab of ZIF-8, generated computationally, immersed in water at 20 and 25 MPa. These two specific values were taken from the intrusion pressures of macro and nanoZIF-8 at 298K presented in figure 2 of the manuscript (the temperature was set to 300K for the simulations). Experimentally, it is impossible to disentangle crystallite size from the pressure at which the heat of intrusion is measured, because the former determines the pressure at which intrusion occurs during experiments (on the timescale accessible in the lab). However, within simulations this disentanglement is possible using special techniques – so-called advanced sampling techniques described in the Supporting Information. In this amended version of the manuscript, the rationale behind the modelling approach is discussed in the main text. Moreover, in the Supporting Information we added more detail on the computational sample. We are confident this will help readers to understand our simulation approach.

c) The paper omits crucial details about water adsorption in nano and macro ZIF-8, such as hydrogen bond networks and adsorption sites, along with the associated insights these details might provide.

We thank the reviewer for drawing our attention to these points, which are very important and, as the comment of the reviewer suggests, require some clarification. At variance with "structural water" present in many crystals, water in ZIF-8 does not have specific absorption sites. Indeed, like the bulk case, water confined in ZIF-8 presents two states: vapor-like and bulk-like.[3,4] *XRD analyses show that there are no key differences between nano- and macroZIF-8 (see figure S1 and table S1 of the updated supporting information file). Indeed, as discussed in previous articles,*[1,2] *the main differences are the intrusion pressure and the intrusion volume. This is due to a different ratio*

between the external surface of the MOF's crystallites and the specific volume of nanoZIF-8 and macroZIF-8 (see the response to point b)).

The lower intrusion pressure in nanoZIF-8 results in a lower intrusion volume, both because of the "pre-wet" incomplete surface cages discussed in point b), but also because the number of water molecules per cage is lower at lower intrusion pressure (figure S10 in the Supporting Information, where the free energy minimum at 20MPa is at a lower number of water molecules (37) than at 25MPa (39)); the density of the intruded water is lower. This change of density obtained from simulations is about one order of magnitude larger than the corresponding change of density of bulk water when one increases the pressure from 20 to 25 MPa (0.6% from data available on the NIST website).

Simulations show that this large difference of density of confined water is responsible for the sizable change of the heat of intrusion between nanoZIF-8 and macroZIF-8. In the amended version of the manuscript, we discuss both the origin of the variation of the intrusion pressure and the consequences on the density of confined water. We remark that already in the original version, simulations were performed with the purpose of explaining the effect of liquid intruding at different pressures.

In light of these issues, I recommend a major revision for this manuscript to address these gaps in methodology and analysis.

Additional Questions:

Urgency: High

Significance: High

Novelty: High

Scholarly Presentation: Moderate

Is the paper likely to interest a substantial number of physical chemists, not just specialists working in the authors' area of research?: Yes

Reviewer: 2

Recommendation: This paper may be publishable, but major revision is needed; I would like to be invited to review any future revision.

Comments:

In this manuscript, the heats of intrusion-extrusion into/from ZIF-8 + water systems of various ZIF-8 crystallite sizes were measured at different temperatures. Their results show that the absolute heat values for water intrusion-extrusion into/out of ZIF-8 are dependent on crystallite size. However, the heat/work ratio of the intrusion-extrusion processes was dependent on temperature whilst independent of crystallite size. The results reported here are interesting and important. However, I have several comments and suggestions outlined below, which should be addressed.

1. P6 L22-23: "At 338.15 K, intrusion was athermic (Qint ≃ 0) and extrusion was exothermic (Qext < 0)." I cannot understand why does the extrusion have quite different heat effect with its reverse process, intrusion?

We thank the reviewer for their question, which we answer below:

Using macroscopic terminology (that is not fully valid for micropores) for the sake of simplicity, we note that intrusion is governed by capillary pressure, whereas extrusion is a thermally activated process governed by bubble nucleation within the cavities[5]. Although these processes are complimentary, they follow distinct mechanisms that influence the output energies. With these factors considered, we hope to have justified the difference in absolute heats related to intrusion-extrusion. We also highlight that the hysteresis in both mechanical energy (difference between intrusion and extrusion pressures) and thermal energy (difference between heat of intrusion and heat of extrusion) is evident at all the recorded temperatures: 338.15K is a peculiar case where the heat of intrusion is negligible.

2. P7 L14-15: "at very high temperature, the intrusion and extrusion pressures of macroZIF-8 decreased". More discussion should be given to explain the underlying mechanism.

This is most likely due to the reduction of contact angle between hydrophobic surfaces and water at higher temperatures. Of course, this is a macroscopic concept, but it explains the relationship between temperature and intrusion pressure: it is easier to wet the pores at higher temperature. In addition, other effects such as bubble nucleation and the energy required to confine water molecules at higher temperatures must be considered to rationalise this initial increase in $P_{int/ext}$ followed by the subsequent decrease. The explanation of such a non-monotonic behaviour is not trivial and was recently discussed in a separate paper[6], where the Cu_2(tebpz) MOF was studied at various temperatures. A similar trend of intrusion pressure and temperature was demonstrated, which was

rationalised by the surprisingly high vapour pressure within the MOF in combination with a reduction in the surface tension of water at the pore aperture at high temperatures.

3. Detailed pore structures of two ZIF-8 samples should be provided.

Both samples have a SOD topology, and very similar structure between them. The key difference is only the crystallite size. The cornerstone of this paper, based on the findings of previous papers of some of the authors of this work, is to clarify the deceptive origin of the thermal effect: this is not due to a different atomistic arrangement but by the effect of crystallite size on the intrusion/extrusion pressures (explained in Johnson et al.[2]) and the ensuing reduction of number of water molecules in the ZIF-8 cages at the lower intrusion pressure. From the XRD patterns and LeBail refinement presented in figure S1 and table S1 of the updated Supporting Information file, we show that there are negligible differences in lattice parameters, yet a significant difference in crystallite size.

4. Are there any differences in chemical composition of two ZIF-8 samples?

We thank the reviewer for this question. There are not. Several batches of nanoZIF-8 were synthesised over the course of this work, of which earlier batches were characterised by NMR and Raman spectroscopy. We saw no differences between these samples and the commercial ZIF-8 purchased from Merck and designated within this work "macroZIF-8". The batch used for the calorimetry measurements had similar intrusion-extrusion characteristics and crystallite structures as determined by XRD to other batches of nanoZIF-8 (equivalent to macroZIF-8 except for peak broadening related to crystallite size).

5. Where is the difference in particle size from? TEM images of two samples should be given to illustrate the difference in particle size.

We thank the reviewer for suggesting these additions, which have now been included in the latest version of the supplementary file (figure S6) in addition to the previously included histogram, which is now figure S7.

6. In Abstract, it claims that These results were mirrored in atomistic simulations, where the reduction of intrusion heat by reducing crystallite size was comparable to the values obtained experimentally. Simulation details should be given to describe how they define the particle sizes of two samples.

We would like to thank the reviewer for drawing our attention to the lack of clarity on this point, confirmed by a corresponding question from reviewer one. For the sake of brevity imposed by a letter, in the original version of this manuscript the presentation of the modeling principles adopted in this work was done mainly via *reference to two articles we published in 2023.[1,2] Your comments, along with those of reviewer one, convinced us that this was insufficient and in this amended version of the manuscript we discuss this in more detail.*

Considering the size of experimental crystallite, 35 nm for nanoZIF-8 and 100 nm or more for the macro counterpart, a brute force simulation approach, with the computational sample consisting of a crystallite of realistic size immersed in sufficient water would be computationally unfeasible. For this reason, we opted for an alternative approach based on the findings of two previous articles,[1,2] *where we showed that the intrusion/extrusion pressures grow with crystallite size despite no significant change in the structure of the MOF (figure S1 and table S1 of the Supporting Information of this amended version of the manuscript). In these articles, we showed that this relationship between intrusion pressure and ZIF-8 crystallite size is related to the external surface area to volume ratio, the latter of which is itself dependent upon crystallite size. This, together with i) the formation of hydrogen bonds between water molecules across hexagonal apertures of the ZIF-8 cavities that reduce the instability of the intruded state, and ii) the incomplete semi-cavities on the surface that are exposed to the bulk liquid and are wet at atmospheric pressure, causes the intrusion pressure of nanoZIF-8 to be approximately 5 MPa lower than in the corresponding macroZIF-8 sample. In practice, the larger surface area/volume ratio reduces the intrusion free energy barrier, and thus nanoZIF-8 can be intruded at lower pressures.*

Based on the findings of these previous articles, summarized in the previous paragraph, here the size of the crystallite is not considered directly but rather by determining the heat of intrusion in a slab of ZIF-8, generated computationally, immersed in water at 20 and 25 MPa. These two specific values were taken from the intrusion pressures of macro and nanoZIF-8 at 298K presented in figure 2 of the manuscript (the temperature was set to 300K for the simulations). Experimentally, it is impossible to disentangle crystallite size from the pressure at which the heat of intrusion is measured, because the former determines the pressure at which intrusion occurs during experiments (on the timescale accessible in the lab). However, within simulations this is possible using special techniques – so-called advanced sampling techniques described in the Supporting Information. In this amended version of the manuscript. the rationale behind the modelling approach is discussed in the main text. Moreover, in the Supporting Information we added more detail on the computational sample. We are confident this will help readers to understand our simulation approach.

Additional Questions:

Urgency: Moderate

Significance: High

Novelty: Moderate

Scholarly Presentation: High

Is the paper likely to interest a substantial number of physical chemists, not just specialists working in the authors' area of research?: Yes

Bibliography

(1) Johnson, L. J. W.; Paulo, G.; Bartolomé, L.; Amayuelas, E.; Gubbiotti, A.; Mirani, D.; Le Donne, A.; López, G. A.; Grancini, G.; Zajdel, P.; Meloni, S.; Giacomello, A.; Grosu, Y. Optimization of the Wetting-Drying Characteristics of Hydrophobic Metal Organic Frameworks via Crystallite Size: The Role of Hydrogen Bonding between Intruded and Bulk Liquid. *Journal of Colloid and Interface Science* **2023**, *645*, 775–783. https://doi.org/10.1016/j.jcis.2023.04.059.

(2) Johnson, L. J. W.; Mirani, D.; Le Donne, A.; Bartolomé, L.; Amayuelas, E.; López, G. A.; Grancini, G.; Carter, M.; Yakovenko, A. A.; Trump, B. A.; Meloni, S.; Zajdel, P.; Grosu, Y. Effect of Crystallite Size on the Flexibility and Negative Compressibility of Hydrophobic Metal–Organic Frameworks. *Nano Lett.* **2023**, *23* (23), 10682–10686. https://doi.org/10.1021/acs.nanolett.3c02431.

(3) Amayuelas, E.; Tortora, M.; Bartolomé, L.; Littlefair, J. D.; Paulo, G.; Le Donne, A.; Trump, B.; Yakovenko, A. A.; Chorążewski, M.; Giacomello, A.; Zajdel, P.; Meloni, S.; Grosu, Y. Mechanism of Water Intrusion into Flexible ZIF-8: Liquid Is Not Vapor. *Nano Lett.* **2023**, *23* (12), 5430–5436. https://doi.org/10.1021/acs.nanolett.3c00235.

(4) Merchiori, S.; Le Donne, A.; Littlefair, J. D.; Lowe, A. R.; Yu, J.-J.; Wu, X.-D.; Li, M.; Li, D.; Geppert-Rybczyńska, M.; Scheller, L.; Trump, B. A.; Yakovenko, A. A.; Zajdel, P.; Chorążewski, M.; Grosu, Y.; Meloni, S. Mild-Temperature Supercritical Water Confined in Hydrophobic Metal–Organic Frameworks. *J. Am. Chem. Soc.* **2024**, *146* (19), 13236–13246. https://doi.org/10.1021/jacs.4c01226.

(5) Lefevre, B.; Saugey, A.; Barrat, J. L.; Bocquet, L.; Charlaix, E.; Gobin, P. F.; Vigier, G. Intrusion and Extrusion of Water in Hydrophobic Mesopores. *The Journal of Chemical Physics* **2004**, *120* (10), 4927–4938.

(6) Merchiori, S.; Donne, A. L.; Bhatia, R.; Alvelli, M.; Yu, J.-J.; Wu, X.-D.; Li, M.; Li, D.; Scheller, L.; Lowe, A. R.; Geppert-Rybczynska, M.; Trump, B. A.; Yakovenko, A. A.; Chorążewski, M.; Zajdel, P.; Grosu, Y.; Meloni, S. Counterintuitive Trend of Intrusion Pressure with Temperature in the Hydrophobic Cu2(Tebpz) MOF. *Small* **2024**, *20* (42), 2402173. https://doi.org/10.1002/smll.202402173.

jz-2024-02639p.R2

Name: Peer Review Information for "Crystallite Size Effects on the Heat of Water Intrusion/Extrusion into/from Metal-organic Frameworks"

Second Round of Reviewer Comments

Reviewer: 1

Comments to the Author

I agree that the authors have properly addressed the previously raised issues. I recommend that the article can be accepted for publication.

Reviewer: 2

Comments to the Author

My only comment is that detailed pore structures of two ZIF-8 samples should be provided.

Author's Response to Peer Review Comments:

Reviewer: 1

Recommendation: This paper represents a significant new contribution and should be published as is.

Comments:
I agree that the authors have properly addressed the previously raised issues. I recommend that the article can be accepted for publication.

Additional Questions:
Urgency: High

Significance: High

Novelty: Top 10%

Scholarly Presentation: High

Is the paper likely to interest a substantial number of physical chemists, not just specialists working in the authors' area of research?: Yes

Reviewer: 2

Recommendation: This paper is publishable subject to minor revisions noted. Further review is not needed.

Comments:
My only comment is that detailed pore structures of two ZIF-8 samples should be provided.

Additional Questions:
Urgency: Moderate

Significance: Moderate

Novelty: High

Scholarly Presentation: High

Is the paper likely to interest a substantial number of physical chemists, not just specialists working in the authors' area of research?: Yes

Once again, we thank the reviewers for their investment of time and e ort in the review of this publication.

For the concern raised by reviewer 2, we have altered the figure S1 in the supporting information relating to the XRD analysis. We have included the di ractograms of the macro and nano ZIF-8 samples along with the simulated di ractogram and structure, the latter rendered in VESTA from the CIF file.[1] As can be seen from the size and strain parameters listed in table S1, the di erence in lattice parameter is minimal (0.01A). Although there are some key di erences between the macro and nano samples (peak broadening related to crystallite size, amorphous contribution most likely due to the grease used during sample preparation or possible disorder within the nano ZIF-8 sample), they both match well with the simulated pattern, reinforcing our reasoning that the two structures are essentially identical, albeit with a significant di erence in crystallite size.

(1) Fairen-Jimenez, D.; Moggach, S. A.; Wharmby, M. T.; Wright, P. A.; Parsons, S.; Düren, T. Opening the Gate: Framework Flexibility in ZIF-8 Explored by Experiments and Simulations. Journal of the American Chemical Society 2011, 133 (23), 8900– 8902. https://doi.org/10.1021/ja202154j.
